# Unusual Clinical Manifestations of *Leishmania (L.) infantum chagasi* in an HIV-coinfected Patient and the Relevance of ITS1-PCR-RFLP: A Case Report

**Published:** 2018

**Authors:** De Godoy NATALIA SOUZA DE, Aiello VERA DEMARCHI, de Souza REGINA MAIA, Okay THELMA, Braz LUCIA MARIA ALMEIDA

**Affiliations:** 1. Laboratory of Investigation in Medical Parasitology, Clínicas Hospital, Faculty of Medicine, University of São Paulo, São Paulo, Brazil; 2. Dept. of Infectious and Parasitic Diseases, Clínicas Hospital, Faculty of Medicine, University of São Paulo, São Paulo, Brazil; 3. Laboratory of Pathological Anatomy, Hearth Institute, Faculty of Medicine, University of São Paulo, São Paulo, Brazil; 4. Laboratory of Seroepidemiology and Immunobiology, Faculty of Medicine, University of São Paulo, São Paulo, Brazil; 5. Laboratory of Parasitology, Institute of Tropical Medicine, University of São Paulo, São Paulo, Brazil

**Keywords:** Visceral leishmaniasis, HIV coinfeccion, Mucocutaneous manifestation, *Leishmania (L.) infantum chagasi*, RFLP

## Abstract

Patients coinfected with *Leishmania*/HIV can develop atypical forms of visceral leishmaniasis (VL), making it indispensable to identify the etiological agent. We are presenting a post-mortem specie definition by ITS1-PCR-RFLP in a larynx tissue of a patient presented coinfection *Leishmania*/HIV. This patient was from a leishmaniasis endemic region in São Paulo (SP), Brazil, and was diagnosed clinically with mucocutaneous leishmaniasis. Before a rK39 immunochromatographic test positive, a tiny stored paraffin-embedded larynx tissue was obtained post-mortem and submitted to 3 conventional PCR assays: kDNA (K20/K22 and RV1/RV2), and ITS1 (LITSR/L5.8S). The last one was followed by RFLP (*Hae*III) and analyzed by 4% Metaphor agarose gel electrophoresis. *Leishmania* genus and *Leishmania* (*Leishmania*) subgenus were defined by kDNA-PCR, with K20/K22 (120 bp) and RV1/RV2 (145 bp), respectively. ITS1-PCR-RFLP identified *L. (L.) infantum chagasi* species visualized by the restriction patterns of 180, 70 and 50 bp. This case draws attention to the necessity for a clear identification of the etiological agent causing infection, especially in endemic regions of cutaneous and visceral leishmaniasis, and particularly in patients with comorbidities who often present atypical forms of the disease. *L.* (*L.*) *infantum chagasi*, which is usually responsible for VL, had changed its clinical spectrum for mucocutaneous. Unequivocal identification was carried out by ITS-PCR-RFLP, therefore confirming rK39 result. These techniques, which complemented each other, have a convenient cost-benefit ratio that makes them suitable to be applied in developing countries.

## Introduction

Visceral leishmaniasis (VL) can be caused mainly by the species of the *donovani* complex, which are *Leishmania donovani* and *L*. (*L*.) *infantum chagasi* (1, 2). In the New World, *L.* (*L.*) *infantum chagasi* is responsible for VL whose most important signs/symptoms include hepatomegaly, splenomegaly, and fever. Leishmaniasis is emerging as the third most frequent opportunistic infection in AIDS patients in several parts of the world, particularly in endemic countries (3). VL/HIV coinfections are associated with atypical forms of VL, dissemination of disease leading to gastric, duodenal, pancreatic, rectal, laryngeal, pulmonary and cutaneous involvement (4). This wide range of clinical presentations turns the identification of the etiological agent necessary and PCR (kDNA and ITS1-RFLP) can solve this. Amplification by ITS1-RFLP is able to differentiate phylogenetically-related organisms (5). However for VL diagnosis in field studies is widely used rK39 immunochromatographic test that uses a recombinant peptide containing 39 amino acid repeats from the kinesin-like gene found in *L. chagasi* (6). Therefore, serological (rK39) and molecular (kDNA and ITS1-RFLP) tests could be used together to define a suspected VL case.

## Case presentation

**Fig. 1:**
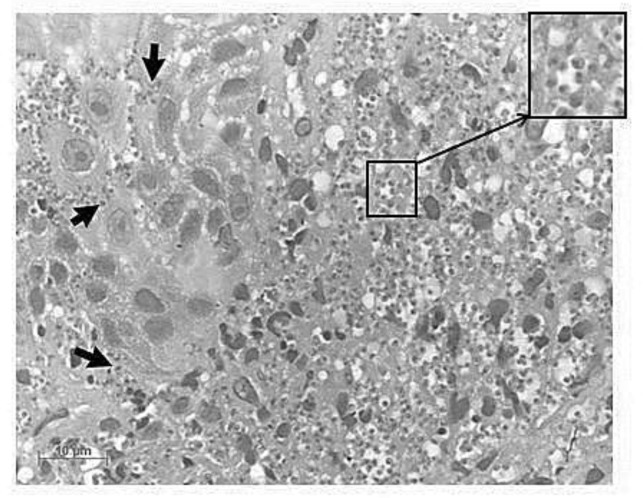
Photomicrograph of the transition between the epithelium and the lamina propria of the vocal fold mucosa. The presence of numerous amastigote forms of *Leishmania* within the cytoplasm of macrophages in the lamina propria, or free parasites among the squamous epithelial cells are evidenced by the arrows. Hematoxylin-eosin staining, magnification 100X

**Fig. 2:**
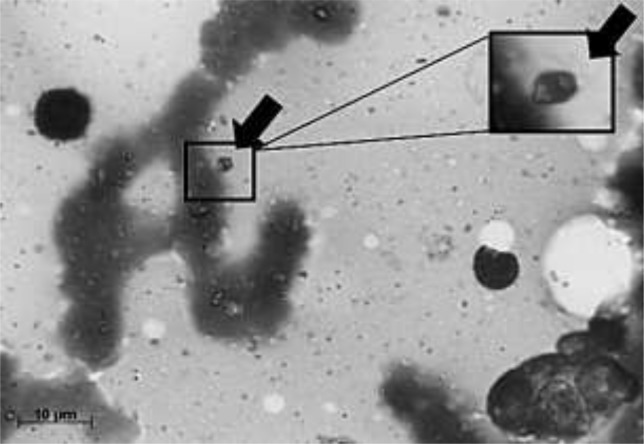
Presence of a *Leishmania* amastigote (arrow) in a Giemsa-stained bone marrow aspirate examined by direct microscopy (magnification 100X)

In 2009, a 47 yr-old man, who lived and worked in the city of Pereira Barreto, state of São Paulo, Brazil, located 650 km from the state capital, received an initial diagnosis of mucocutaneous leishmaniasis (ML) affecting his face and oral mucosa. His hometown belongs to an area in which there is a high transmission of cutaneous (CL) and visceral leishmaniasis (VL) in dogs and humans (7, 8). The patient was successfully treated with meglumine antimoniate for a month (1200 mg/day, intramuscular). Two yr later, in 2011, he attended the same outpatient clinic presenting a significant weight loss and mucocutaneous lesions located at the same region of the ones diagnosed in 2009. Once again he was successfully treated with meglumine antimoniate for a month (same therapeutic regimen). The patient was also diagnosed with HIV and hepatitis C (high viral loads) and had a CD4+ cell count of 198/mm^3^. An antiretroviral treatment (HAART) was started, with significant viral load reduction, but soon after, the medical monitoring was abandoned again, when he was discharged from hospital. Patient’s last hospital admission occurred in Jul 2013. He was attended at the *Hospital das Clinicas da Faculdade de Medicina da Universidade de São Paulo* (HCFMUSP), a tertiary reference center located in the city of São Paulo (SP). A severe hoarseness, discomfort swallowing, and whitish oropharyngeal lesions, aside from episodes of cough, fever, sweating and a marked weight loss referred to in the last two months were presented by the patient. In addition, the abdominal ultrasonography revealed the presence of a marked splenomegaly. The CD4+ cell count was 40/mm^3^ and the HIV and hepatitis C viral loads were extremely high. Due to the severe hoarseness, a larynx biopsy was performed and the histological findings (Fig. 1) confirmed the diagnosis of ML. In this time, a Visceralization of the ML had occurred because of a pancytopenia and the presence of *Leishmania* amastigotes in stained smears from a bone marrow aspirate (Fig. 2). Regarding the leishmaniasis, the patient received 200 mg/day of intravenous liposomal amphotericin B for 10 d. Before another myelogram cytomegalo-virus-infected cells were evidenced and treated with ganciclovir (5 mg/kg, twice a day, 21 d). As the oropharyngeal lesions did not improve, liposomal amphotericin B was reintroduced (same therapeutic regimen). The patient evolved with temporary hemodynamic stability and a Glasgow coma scale of 14 and developed respiratory distress and tachypnea. Although being treated with broad-spectrum antibiotics, ganciclovir, and liposomal amphotericin B, the patient progressed with clinical deterioration and died after 75 d of hospitalization. According to the medical chart, the final diagnosis was a ML visceralized due to the presence of numerous comorbidities such as the HIV and the hepatitis C infection.

Serological techniques performed at the end of the patient life were positive (rK39 immunochromatographic test - Kalazar DetectTM -InBios, Inc., Seattle, WA, USA; Indirect Immunofluorescence-IFA -Biomanguinhos® and an in-house ELISA) (9). After the analysis of the patient’s medical chart, the following things have caught our attention: the patient’s hometown is a known endemic area for both, CL and VL, as well as, the initially unknown HIV coinfection and the positivity of serological tests (mainly rK39). Therefore, we decided to investigate the etiological agent responsible for these clinical manifestations

This study was approved by the institutional Ethics Committee (CAPPesq) process number 0006/11.

An extremely tiny stored paraffin-embedded larynx biopsy was retrieved from Pathology Laboratory. After DNA extraction it was submitted to 3 conventional PCR assays: kDNA (K20/K22) (2) and (RV1/RV2) (10) and ITS1 (LITSR/L5.8S) (11, 12). Amplifications have followed previously described protocols (2, 12), with strict measures to minimize the risk of carry-over contamination (2, 12, 13). Reference strains, that occurs in Brazil, obtained from cultures, were used as positive controls: *L.* (*L.*) *chagasi* (MHOM/BR/81/M6445), *L.* (*L.*) *amazonensis* (MHOM/BR/1973/M2269), *L.* (*Viannia*) *guyanensis* (MHOM/BR/1975/M4147) and *L.*(*V.*) *braziliensis* (MHOM/BR/75/M2903).

**Fig. 3:**
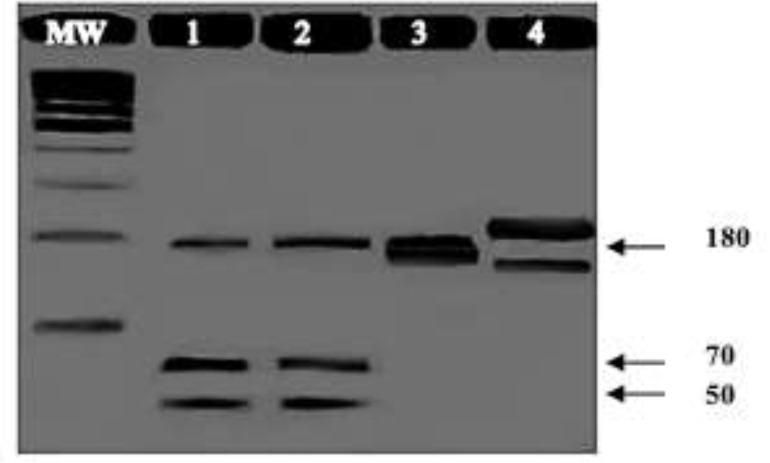
ITS1-PCR-RFLP (*Hae*III) patterns of prototype *Leishmania* species and the patient sample. The molecular weight marker (MW) is the 100 bp ladder. 1- the patient sample; 2- *L.* (*L.*) *chagasi* (MHOM/BR/81/M6445); 3- *L.* (*V*) *braziliensis* (MHOM/BR/75/M2903); 4- *L.* (*L.*) *amazonensis* (MHOM/BR/1973/M2269)

*Leishmania* genus was defined by kDNAK20/K22 (120 bp) and ITS1 (320bp), and subgenus by RV1/RV2 (145 bp). *L.* (*L.*) *infantum chagasi* specie was identified by the ITS1-RFLP (180, 70 and 50 bp) (Fig. 3).

## Discussion

A peculiarity of the coinfection leishmaniasis/HIV, even in the HAART era, is tendency to relapse (25%–61%), normally within one year, (14) additionally to a high mortality rate, and the occurrence of unusual clinical manifestations. From 2009, our patient had many episodes of ML relapses. Regarding the tropism of *Leishmania*, many authors have reported them in unusual locations, although they have not always been able to evidence the etiological agent (1, 3, 4, 15). Serological tests allowed the rapid detection of anti-*Leishmania* antibodies in cases of clinical suspect of VL. However, before unusual symptoms, it is not so common the use of such methods. In patients with HIV, mainly in areas endemic for VL, the use of serological methods should be routinely performed (16).

In a rare case of VL/HIV infection in which there were pulmonary and oral lesions, the etiological agent was defined as *L.* (*L.*) *infantum,* zimodeme MON1, characterized by Multiloccus Enzyme Electroforesis (MLEE) (17). In our case, the identification of *L.* (*L.*)*. infantum chagasi* was accomplished by PCR-ITS1-RFLP in a stored larynx biopsy fragment and the result of this analysis pointed out an unusual location of the parasite. In addition, RFLP has the advantage of not requiring live parasites as does MLEE. Unfortunately, the identification *of L.* (*L.*) *infantum chagasi* took place only *post-mortem* and maybe if it had been happen when the patient was alive, the treatment could have been different. Nevertheless, the ITS1-PCR-RFLP identified *L.* (*L*.)*. infantum chagasi* as the etiological agent in this patient, therefore corroborating the finding of serology (positive rK39), an immunochromatographic test that is specific for the *Leishmania donovani* complex, which includes *L.* (*L.*) *infantum chagasi*. Moreover, by means of the ITS-PCR-RFLP (11), our group has recently identified *L. infantum chagasi* in a rare case of PKDL in a VL/HIV coinfected patient (13). *L. tropica*, a specie involved in cutaneous leishmaniasis (CL) in Iran, was found to be responsible for unusual manifestations in patients coinfected with *Leishmania*/HIV, who presented skin lesions with *Leishmania* bodies and parasites in viscera detected by RAPDPCR technique, confirmed by PCR-RFLP (1). A case of a patient was reported with VL presenting clinical manifestation of ML and CL, with skin and oral lesions, in which the nested PCR, followed by confirmation through sequencing, were used and allowed the definition of *L. major* as an etiological agent of the presented clinical condition (15). As the present study, serology had great value in another study (15), because IFAT and direct agglutination test (DAT) were also positive and presented high titers indicating visceral involvement.

## Conclusion

Our report paid attention to the need for a clear identification of the etiological agent and the species causing infection, especially in endemic regions of CL and VL, particularly in patients with comorbidities, who often present atypical forms of the disease. ITS1-PCR-RFLP identified *L. (L.). infantum chagasi* as the etiological agent of this ML, corroborating the finding of serology (positive rK39), specific for the *Leishmania donovani* complex. Additionally, kDNA, rk39, and ITS1-RFLP have a convenient cost-benefit ratio that makes them suitable to be applied in developing countries.
